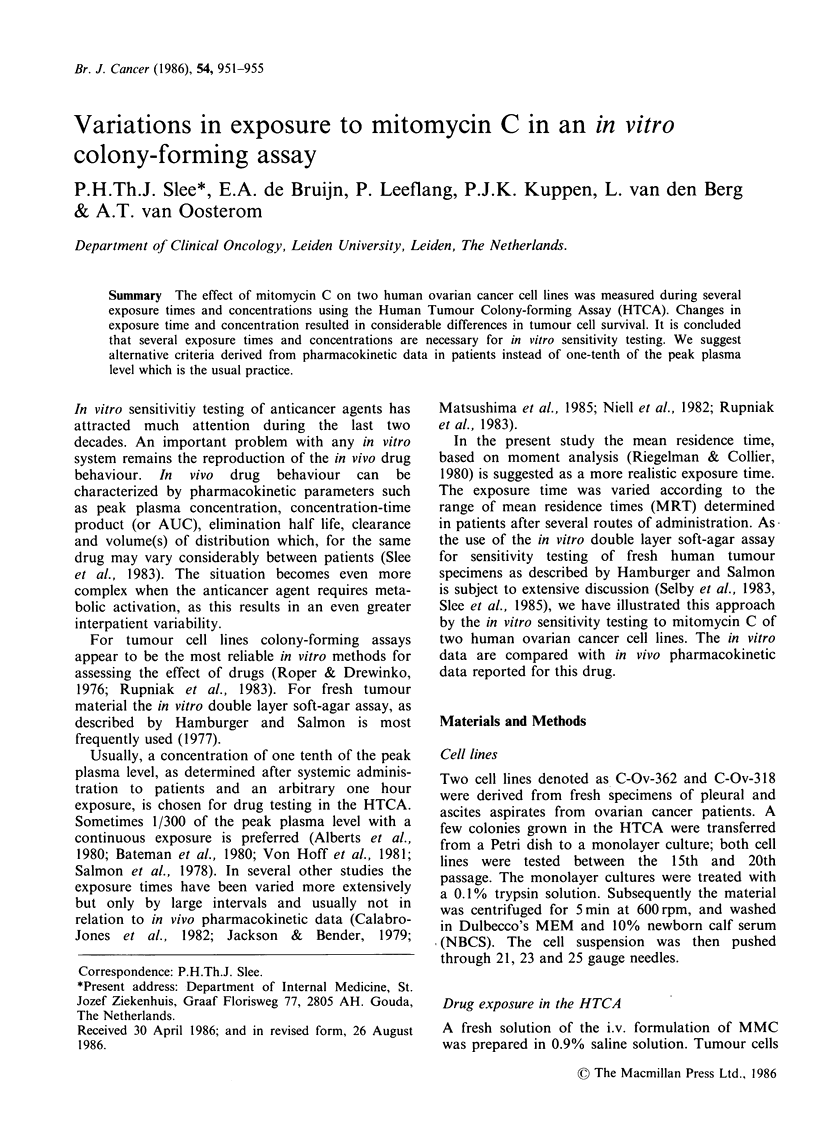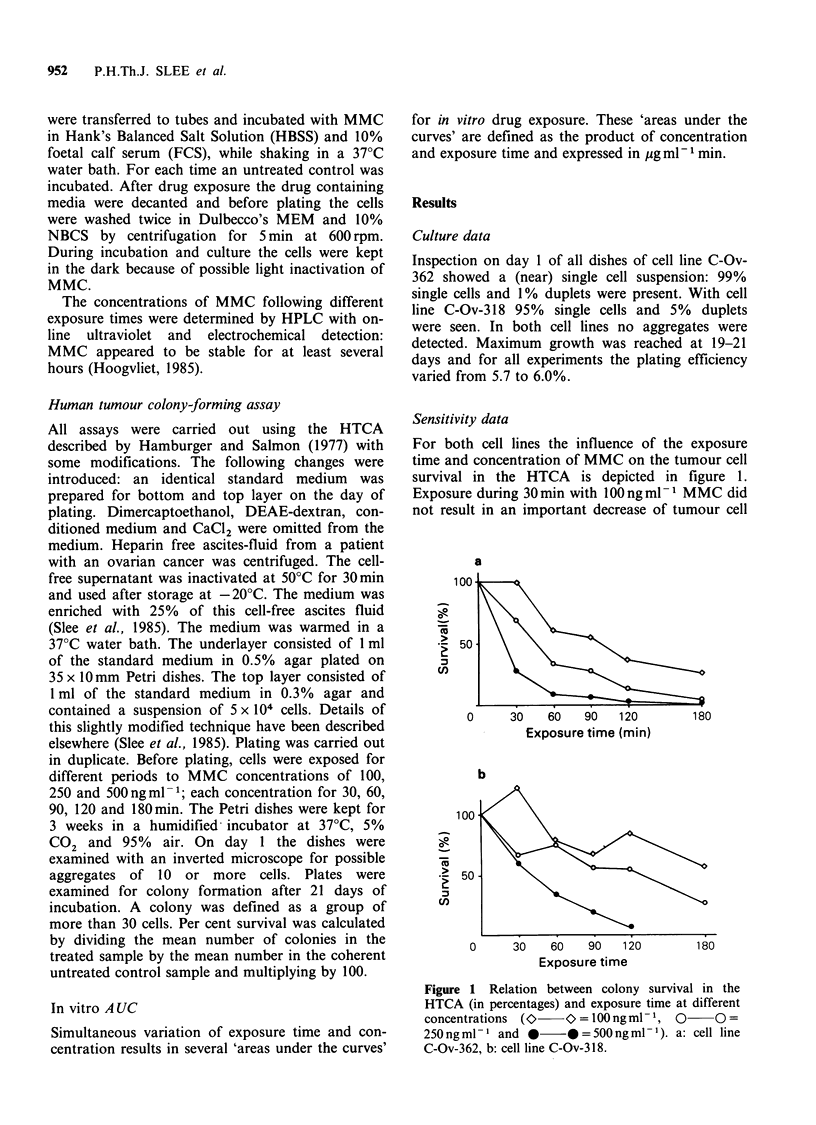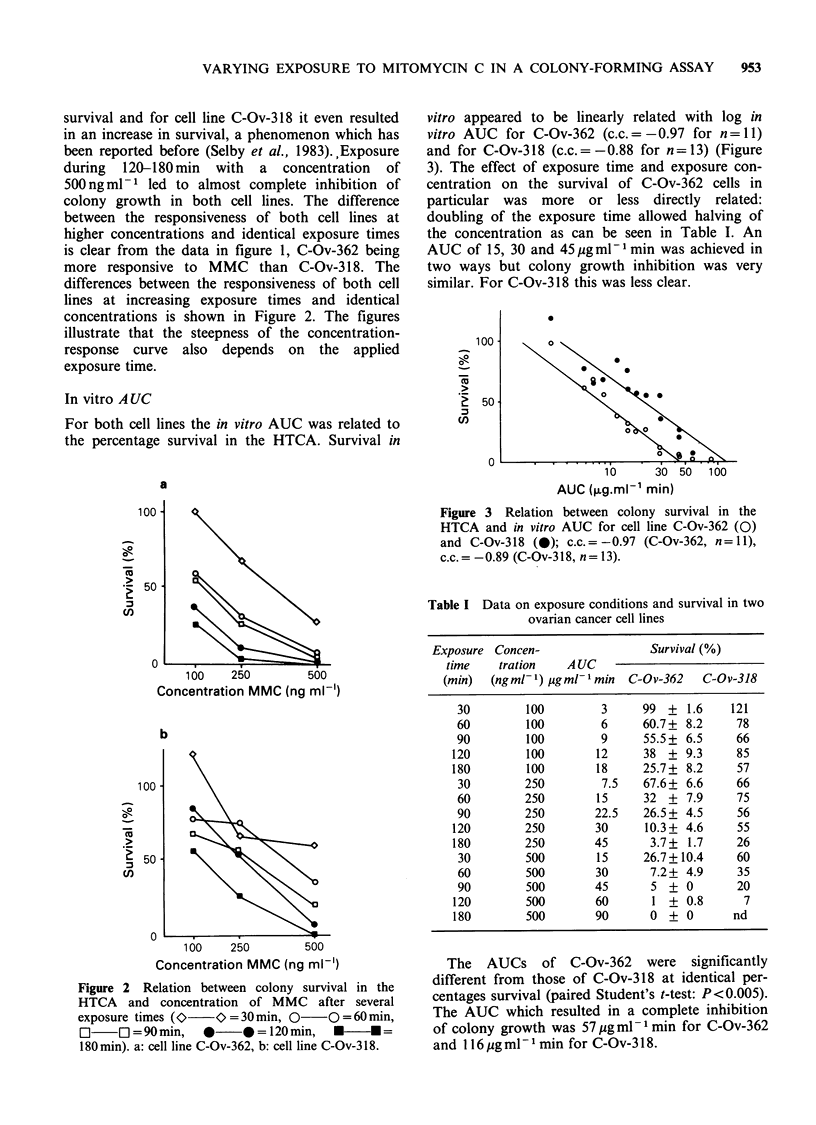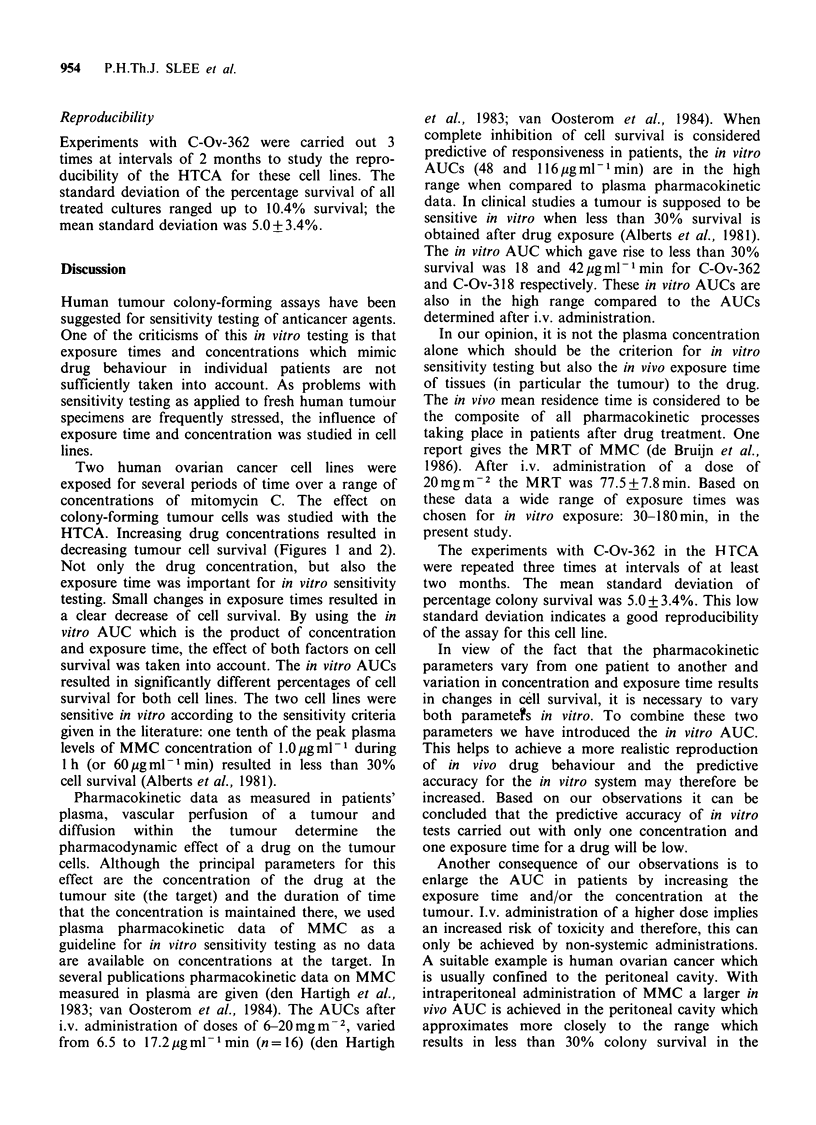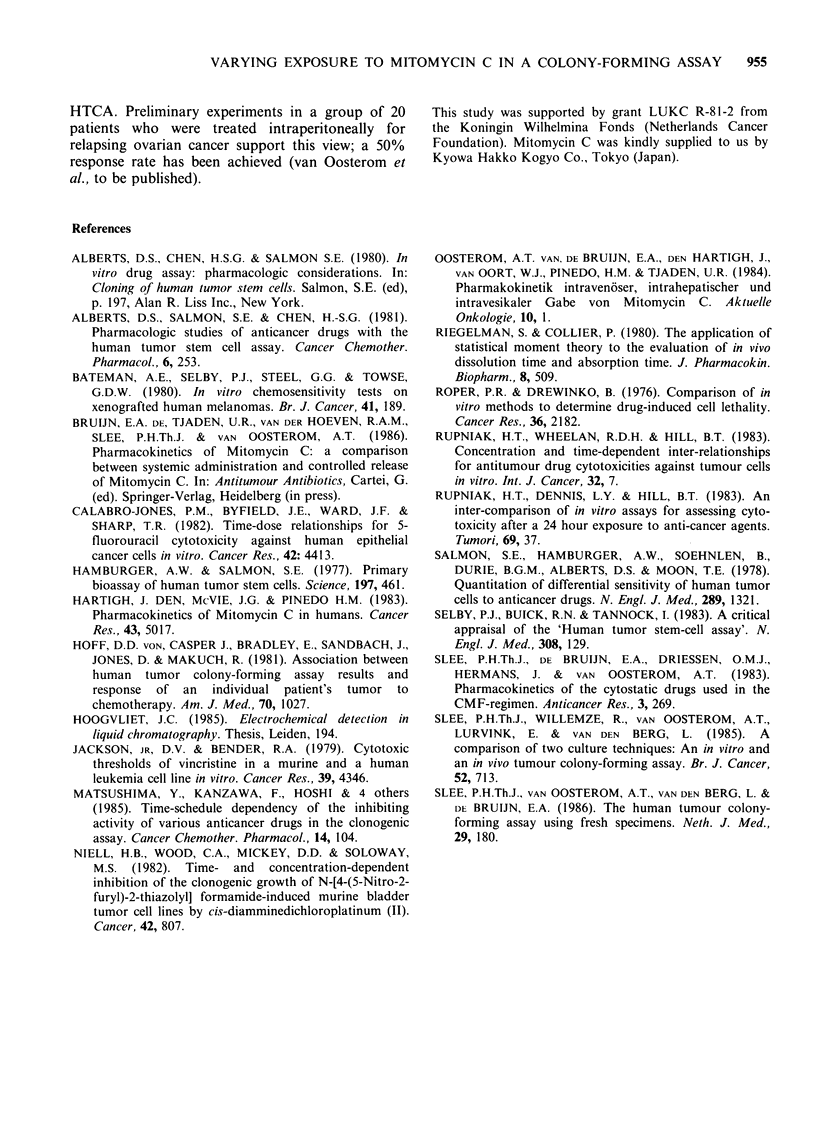# Variations in exposure to mitomycin C in an in vitro colony-forming assay.

**DOI:** 10.1038/bjc.1986.266

**Published:** 1986-12

**Authors:** P. H. Slee, E. A. de Bruijn, P. Leeflang, P. J. Kuppen, L. van den Berg, A. T. van Oosterom

## Abstract

The effect of mitomycin C on two human ovarian cancer cell lines was measured during several exposure times and concentrations using the Human Tumour Colony-forming Assay (HTCA). Changes in exposure time and concentration resulted in considerable differences in tumour cell survival. It is concluded that several exposure times and concentrations are necessary for in vitro sensitivity testing. We suggest alternative criteria derived from pharmacokinetic data in patients instead of one-tenth of the peak plasma level which is the usual practice.


*Br. J. Cancer* (1986), **54**, 951–955

### Variations in exposure to mitomycin C in an *in vitro* colony-forming assay

P.H.Th.J. Slee*, E.A. de Bruijn, P. Leeflang, P.J.K. Kuppen, L. van den Berg & A.T. van Oosterom

*Department of Clinical Oncology, Leiden University, Leiden, The Netherlands.*

**Summary** The effect of mitomycin C on two human ovarian cancer cell lines was measured during several exposure times and concentrations using the Human Tumour Colony-forming Assay (HTCA). Changes in exposure time and concentration resulted in considerable differences in tumour cell survival. It is concluded that several exposure times and concentrations are necessary for *in vitro* sensitivity testing. We suggest alternative criteria derived from pharmacokinetic data in patients instead of one-tenth of the peak plasma level which is the usual practice.

*In vitro* sensitivitiy testing of anticancer agents has attracted much attention during the last two decades. An important problem with any *in vitro* system remains the reproduction of the *in vivo* drug behaviour. *In vivo* drug behaviour can be characterized by pharmacokinetic parameters such as peak plasma concentration, concentration-time product (or AUC), elimination half life, clearance and volume(s) of distribution which, for the same drug may vary considerably between patients (Slee *et al.*, 1983). The situation becomes even more complex when the anticancer agent requires metabolic activation, as this results in an even greater interpatient variability.

For tumour cell lines colony-forming assays appear to be the most reliable *in vitro* methods for assessing the effect of drugs (Roper & Drewinko, 1976; Rupniak *et al.*, 1983). For fresh tumour material the *in vitro* double layer soft-agar assay, as described by Hamburger and Salmon is most frequently used (1977).

Usually, a concentration of one tenth of the peak plasma level, as determined after systemic administration to patients and an arbitrary one hour exposure, is chosen for drug testing in the HTCA. Sometimes 1/300 of the peak plasma level with a continuous exposure is preferred (Alberts *et al.*, 1980; Bateman *et al.*, 1980; Von Hoff *et al.*, 1981; Salmon *et al.*, 1978). In several other studies the exposure times have been varied more extensively but only by large intervals and usually not in relation to *in vivo* pharmacokinetic data (Calabro-Jones *et al.*, 1982; Jackson & Bender, 1979; Matsushima *et al.*, 1985; Niell *et al.*, 1982; Rupniak *et al.*, 1983).

In the present study the mean residence time, based on moment analysis (Riegelman & Collier, 1980) is suggested as a more realistic exposure time. The exposure time was varied according to the range of mean residence times (MRT) determined in patients after several routes of administration. As the use of the *in vitro* double layer soft-agar assay for sensitivity testing of fresh human tumour specimens as described by Hamburger and Salmon is subject to extensive discussion (Selby *et al.*, 1983, Slee *et al.*, 1985), we have illustrated this approach by the *in vitro* sensitivity testing to mitomycin C of two human ovarian cancer cell lines. The *in vitro* data are compared with *in vivo* pharmacokinetic data reported for this drug.

### Materials and Methods

### *Cell lines*

Two cell lines denoted as C-Ov-362 and C-Ov-318 were derived from fresh specimens of pleural and ascites aspirates from ovarian cancer patients. A few colonies grown in the HTCA were transferred from a Petri dish to a monolayer culture; both cell lines were tested between the 15th and 20th passage. The monolayer cultures were treated with a 0.1% trypsin solution. Subsequently the material was centrifuged for 5 min at 600 rpm, and washed in Dulbecco's MEM and 10% newborn calf serum (NBCS). The cell suspension was then pushed through 21, 23 and 25 gauge needles.

### *Drug exposure in the HTCA*

A fresh solution of the i.v. formulation of MMC was prepared in 0.9% saline solution. Tumour cells

Correspondence: P.H.Th.J. Slee.
*Present address: Department of Internal Medicine, St. Jozef Ziekenhuis, Graaf Florisweg 77, 2805 AH. Gouda, The Netherlands.
Received 30 April 1986; and in revised form, 26 August 1986.

© The Macmillan Press Ltd., 1986

were transferred to tubes and incubated with MMC in Hank's Balanced Salt Solution (HBSS) and 10% foetal calf serum (FCS), while shaking in a 37°C water bath. For each time an untreated control was incubated. After drug exposure the drug containing media were decanted and before plating the cells were washed twice in Dulbecco's MEM and 10% NBCS by centrifugation for 5 min at 600 rpm. During incubation and culture the cells were kept in the dark because of possible light inactivation of MMC.

The concentrations of MMC following different exposure times were determined by HPLC with on-line ultraviolet and electrochemical detection: MMC appeared to be stable for at least several hours (Hoogvliet, 1985).

### *Human tumour colony-forming assay*

All assays were carried out using the HTCA described by Hamburger and Salmon (1977) with some modifications. The following changes were introduced: an identical standard medium was prepared for bottom and top layer on the day of plating. Dimercaptoethanol, DEAE-dextran, conditioned medium and $CaCl_2$ were omitted from the medium. Heparin free ascites-fluid from a patient with an ovarian cancer was centrifuged. The cell-free supernatant was inactivated at 50°C for 30 min and used after storage at −20°C. The medium was enriched with 25% of this cell-free ascites fluid (Slee *et al.*, 1985). The medium was warmed in a 37°C water bath. The underlayer consisted of 1 ml of the standard medium in 0.5% agar plated on 35 x 10 mm Petri dishes. The top layer consisted of 1 ml of the standard medium in 0.3% agar and contained a suspension of $5 \times 10^4$ cells. Details of this slightly modified technique have been described elsewhere (Slee *et al.*, 1985). Plating was carried out in duplicate. Before plating, cells were exposed for different periods to MMC concentrations of 100, 250 and 500 ng ml$^{-1}$; each concentration for 30, 60, 90, 120 and 180 min. The Petri dishes were kept for 3 weeks in a humidified incubator at 37°C, 5% $CO_2$ and 95% air. On day 1 the dishes were examined with an inverted microscope for possible aggregates of 10 or more cells. Plates were examined for colony formation after 21 days of incubation. A colony was defined as a group of more than 30 cells. Per cent survival was calculated by dividing the mean number of colonies in the treated sample by the mean number in the coherent untreated control sample and multiplying by 100.

### In vitro *AUC*

Simultaneous variation of exposure time and concentration results in several 'areas under the curves' for *in vitro* drug exposure. These 'areas under the curves' are defined as the product of concentration and exposure time and expressed in $\mu$g ml$^{-1}$ min.

### Results

### *Culture data*

Inspection on day 1 of all dishes of cell line C-Ov-362 showed a (near) single cell suspension: 99% single cells and 1% duplets were present. With cell line C-Ov-318 95% single cells and 5% duplets were seen. In both cell lines no aggregates were detected. Maximum growth was reached at 19–21 days and for all experiments the plating efficiency varied from 5.7 to 6.0%.

### *Sensitivity data*

For both cell lines the influence of the exposure time and concentration of MMC on the tumour cell survival in the HTCA is depicted in figure 1. Exposure during 30 min with 100 ng ml$^{-1}$ MMC did not result in an important decrease of tumour cell

**Figure 1** Relation between colony survival in the HTCA (in percentages) and exposure time at different concentrations (◇——◇ = 100 ng ml$^{-1}$, ○——○ = 250 ng ml$^{-1}$ and ●——● = 500 ng ml$^{-1}$). a: cell line C-Ov-362, b: cell line C-Ov-318.

survival and for cell line C-Ov-318 it even resulted in an increase in survival, a phenomenon which has been reported before (Selby *et al.*, 1983). Exposure during 120–180 min with a concentration of 500 ng ml$^{-1}$ led to almost complete inhibition of colony growth in both cell lines. The difference between the responsiveness of both cell lines at higher concentrations and identical exposure times is clear from the data in figure 1, C-Ov-362 being more responsive to MMC than C-Ov-318. The differences between the responsiveness of both cell lines at increasing exposure times and identical concentrations is shown in Figure 2. The figures illustrate that the steepness of the concentration-response curve also depends on the applied exposure time.

### In vitro *AUC*

For both cell lines the *in vitro* AUC was related to the percentage survival in the HTCA. Survival *in vitro* appeared to be linearly related with log *in vitro* AUC for C-Ov-362 (c.c. = −0.97 for $n=11$) and for C-Ov-318 (c.c. = −0.88 for $n=13$) (Figure 3). The effect of exposure time and exposure concentration on the survival of C-Ov-362 cells in particular was more or less directly related: doubling of the exposure time allowed halving of the concentration as can be seen in Table I. An AUC of 15, 30 and 45 $\mu$g ml$^{-1}$ min was achieved in two ways but colony growth inhibition was very similar. For C-Ov-318 this was less clear.

**Figure 2** Relation between colony survival in the HTCA and concentration of MMC after several exposure times (◇——◇ = 30 min, ○——○ = 60 min, □——□ = 90 min, ●——● = 120 min, ■——■ = 180 min). a: cell line C-Ov-362, b: cell line C-Ov-318.

**Figure 3** Relation between colony survival in the HTCA and *in vitro* AUC for cell line C-Ov-362 (○) and C-Ov-318 (●); c.c. = −0.97 (C-Ov-362, $n=11$), c.c. = −0.89 (C-Ov-318, $n=13$).

**Table I** Data on exposure conditions and survival in two ovarian cancer cell lines

| *Exposure time (min)* | *Concentration (ng ml$^{-1}$)* | *AUC μg ml$^{-1}$ min* | *Survival (%)* C-Ov-362 | *Survival (%)* C-Ov-318 |
|---|---|---|---|---|
| 30 | 100 | 3 | 99 ± 1.6 | 121 |
| 60 | 100 | 6 | 60.7 ± 8.2 | 78 |
| 90 | 100 | 9 | 55.5 ± 6.5 | 66 |
| 120 | 100 | 12 | 38 ± 9.3 | 85 |
| 180 | 100 | 18 | 25.7 ± 8.2 | 57 |
| 30 | 250 | 7.5 | 67.6 ± 6.6 | 66 |
| 60 | 250 | 15 | 32 ± 7.9 | 75 |
| 90 | 250 | 22.5 | 26.5 ± 4.5 | 56 |
| 120 | 250 | 30 | 10.3 ± 4.6 | 55 |
| 180 | 250 | 45 | 3.7 ± 1.7 | 26 |
| 30 | 500 | 15 | 26.7 ± 10.4 | 60 |
| 60 | 500 | 30 | 7.2 ± 4.9 | 35 |
| 90 | 500 | 45 | 5 ± 0 | 20 |
| 120 | 500 | 60 | 1 ± 0.8 | 7 |
| 180 | 500 | 90 | 0 ± 0 | nd |

The AUCs of C-Ov-362 were significantly different from those of C-Ov-318 at identical percentages survival (paired Student's *t*-test: $P<0.005$). The AUC which resulted in a complete inhibition of colony growth was 57 $\mu$g ml$^{-1}$ min for C-Ov-362 and 116 $\mu$g ml$^{-1}$ min for C-Ov-318.

*Reproducibility*

Experiments with C-Ov-362 were carried out 3 times at intervals of 2 months to study the reproducibility of the HTCA for these cell lines. The standard deviation of the percentage survival of all treated cultures ranged up to 10.4% survival; the mean standard deviation was $5.0 \pm 3.4\%$.

### Discussion

Human tumour colony-forming assays have been suggested for sensitivity testing of anticancer agents. One of the criticisms of this *in vitro* testing is that exposure times and concentrations which mimic drug behaviour in individual patients are not sufficiently taken into account. As problems with sensitivity testing as applied to fresh human tumour specimens are frequently stressed, the influence of exposure time and concentration was studied in cell lines.

Two human ovarian cancer cell lines were exposed for several periods of time over a range of concentrations of mitomycin C. The effect on colony-forming tumour cells was studied with the HTCA. Increasing drug concentrations resulted in decreasing tumour cell survival (Figures 1 and 2). Not only the drug concentration, but also the exposure time was important for *in vitro* sensitivity testing. Small changes in exposure times resulted in a clear decrease of cell survival. By using the *in vitro* AUC which is the product of concentration and exposure time, the effect of both factors on cell survival was taken into account. The *in vitro* AUCs resulted in significantly different percentages of cell survival for both cell lines. The two cell lines were sensitive *in vitro* according to the sensitivity criteria given in the literature: one tenth of the peak plasma levels of MMC concentration of $1.0\,\mu g\,ml^{-1}$ during 1 h (or $60\,\mu g\,ml^{-1}\,min$) resulted in less than 30% cell survival (Alberts *et al.*, 1981).

Pharmacokinetic data as measured in patients' plasma, vascular perfusion of a tumour and diffusion within the tumour determine the pharmacodynamic effect of a drug on the tumour cells. Although the principal parameters for this effect are the concentration of the drug at the tumour site (the target) and the duration of time that the concentration is maintained there, we used plasma pharmacokinetic data of MMC as a guideline for *in vitro* sensitivity testing as no data are available on concentrations at the target. In several publications pharmacokinetic data on MMC measured in plasma are given (den Hartigh *et al.*, 1983; van Oosterom *et al.*, 1984). The AUCs after i.v. administration of doses of $6–20\,mg\,m^{-2}$, varied from 6.5 to $17.2\,\mu g\,ml^{-1}\,min$ ($n = 16$) (den Hartigh *et al.*, 1983; van Oosterom *et al.*, 1984). When complete inhibition of cell survival is considered predictive of responsiveness in patients, the *in vitro* AUCs (48 and $116\,\mu g\,ml^{-1}\,min$) are in the high range when compared to plasma pharmacokinetic data. In clinical studies a tumour is supposed to be sensitive *in vitro* when less than 30% survival is obtained after drug exposure (Alberts *et al.*, 1981). The *in vitro* AUC which gave rise to less than 30% survival was 18 and $42\,\mu g\,ml^{-1}\,min$ for C-Ov-362 and C-Ov-318 respectively. These *in vitro* AUCs are also in the high range compared to the AUCs determined after i.v. administration.

In our opinion, it is not the plasma concentration alone which should be the criterion for *in vitro* sensitivity testing but also the *in vivo* exposure time of tissues (in particular the tumour) to the drug. The *in vivo* mean residence time is considered to be the composite of all pharmacokinetic processes taking place in patients after drug treatment. One report gives the MRT of MMC (de Bruijn *et al.*, 1986). After i.v. administration of a dose of $20\,mg\,m^{-2}$ the MRT was $77.5 \pm 7.8\,min$. Based on these data a wide range of exposure times was chosen for *in vitro* exposure: 30–180 min, in the present study.

The experiments with C-Ov-362 in the HTCA were repeated three times at intervals of at least two months. The mean standard deviation of percentage colony survival was $5.0 \pm 3.4\%$. This low standard deviation indicates a good reproducibility of the assay for this cell line.

In view of the fact that the pharmacokinetic parameters vary from one patient to another and variation in concentration and exposure time results in changes in cell survival, it is necessary to vary both parameters *in vitro*. To combine these two parameters we have introduced the *in vitro* AUC. This helps to achieve a more realistic reproduction of *in vivo* drug behaviour and the predictive accuracy for the *in vitro* system may therefore be increased. Based on our observations it can be concluded that the predictive accuracy of *in vitro* tests carried out with only one concentration and one exposure time for a drug will be low.

Another consequence of our observations is to enlarge the AUC in patients by increasing the exposure time and/or the concentration at the tumour. I.v. administration of a higher dose implies an increased risk of toxicity and therefore, this can only be achieved by non-systemic administrations. A suitable example is human ovarian cancer which is usually confined to the peritoneal cavity. With intraperitoneal administration of MMC a larger *in vivo* AUC is achieved in the peritoneal cavity which approximates more closely to the range which results in less than 30% colony survival in the

HTCA. Preliminary experiments in a group of 20 patients who were treated intraperitoneally for relapsing ovarian cancer support this view; a 50% response rate has been achieved (van Oosterom *et al.*, to be published).

This study was supported by grant LUKC R-81-2 from the Koningin Wilhelmina Fonds (Netherlands Cancer Foundation). Mitomycin C was kindly supplied to us by Kyowa Hakko Kogyo Co., Tokyo (Japan).

### References

ALBERTS, D.S., CHEN, H.S.G. & SALMON S.E. (1980). *In vitro* drug assay: pharmacologic considerations. In: *Cloning of human tumor stem cells*. Salmon, S.E. (ed), p. 197, Alan R. Liss Inc., New York.

ALBERTS, D.S., SALMON, S.E. & CHEN, H.-S.G. (1981). Pharmacologic studies of anticancer drugs with the human tumor stem cell assay. *Cancer Chemother. Pharmacol.*, **6**, 253.

BATEMAN, A.E., SELBY, P.J., STEEL, G.G. & TOWSE, G.D.W. (1980). *In vitro* chemosensitivity tests on xenografted human melanomas. *Br. J. Cancer*, **41**, 189.

BRUIJN, E.A. DE, TJADEN, U.R., VAN DER HOEVEN, R.A.M., SLEE, P.H.Th.J. & VAN OOSTEROM, A.T. (1986). Pharmacokinetics of Mitomycin C: a comparison between systemic administration and controlled release of Mitomycin C. In: *Antitumour Antibiotics*, Cartei, G. (ed). Springer-Verlag, Heidelberg (in press).

CALABRO-JONES, P.M., BYFIELD, J.E., WARD, J.F. & SHARP, T.R. (1982). Time-dose relationships for 5-fluorouracil cytotoxicity against human epithelial cancer cells *in vitro*. *Cancer Res.*, **42**: 4413.

HAMBURGER, A.W. & SALMON, S.E. (1977). Primary bioassay of human tumor stem cells. *Science*, **197**, 461.

HARTIGH, J. DEN, McVIE, J.G. & PINEDO H.M. (1983). Pharmacokinetics of Mitomycin C in humans. *Cancer Res.*, **43**, 5017.

HOFF, D.D. VON, CASPER J., BRADLEY, E., SANDBACH, J., JONES, D. & MAKUCH, R. (1981). Association between human tumor colony-forming assay results and response of an individual patient's tumor to chemotherapy. *Am. J. Med.*, **70**, 1027.

HOOGVLIET, J.C. (1985). *Electrochemical detection in liquid chromatography*. Thesis, Leiden, 194.

JACKSON, JR, D.V. & BENDER, R.A. (1979). Cytotoxic thresholds of vincristine in a murine and a human leukemia cell line *in vitro*. *Cancer Res.*, **39**, 4346.

MATSUSHIMA, Y., KANZAWA, F., HOSHI & 4 others (1985). Time-schedule dependency of the inhibiting activity of various anticancer drugs in the clonogenic assay. *Cancer Chemother. Pharmacol.*, **14**, 104.

NIELL, H.B., WOOD, C.A., MICKEY, D.D. & SOLOWAY, M.S. (1982). Time- and concentration-dependent inhibition of the clonogenic growth of N-[4-(5-Nitro-2-furyl)-2-thiazolyl] formamide-induced murine bladder tumor cell lines by *cis*-diamminedichloroplatinum (II). *Cancer*, **42**, 807.

OOSTEROM, A.T. VAN, DE BRUIJN, E.A., DEN HARTIGH, J., VAN OORT, W.J., PINEDO, H.M. & TJADEN, U.R. (1984). Pharmakokinetik intravenöser, intrahepatischer und intravesikaler Gabe von Mitomycin C. *Aktuelle Onkologie*, **10**, 1.

RIEGELMAN, S. & COLLIER, P. (1980). The application of statistical moment theory to the evaluation of *in vivo* dissolution time and absorption time. *J. Pharmacokin. Biopharm.*, **8**, 509.

ROPER, P.R. & DREWINKO, B. (1976). Comparison of *in vitro* methods to determine drug-induced cell lethality. *Cancer Res.*, **36**, 2182.

RUPNIAK, H.T., WHEELAN, R.D.H. & HILL, B.T. (1983). Concentration and time-dependent inter-relationships for antitumour drug cytotoxicities against tumour cells *in vitro*. *Int. J. Cancer*, **32**, 7.

RUPNIAK, H.T., DENNIS, L.Y. & HILL, B.T. (1983). An inter-comparison of *in vitro* assays for assessing cytotoxicity after a 24 hour exposure to anti-cancer agents. *Tumori*, **69**, 37.

SALMON, S.E., HAMBURGER, A.W., SOEHNLEN, B., DURIE, B.G.M., ALBERTS, D.S. & MOON, T.E. (1978). Quantitation of differential sensitivity of human tumor cells to anticancer drugs. *N. Engl. J. Med.*, **289**, 1321.

SELBY, P.J., BUICK, R.N. & TANNOCK, I. (1983). A critical appraisal of the 'Human tumor stem-cell assay'. *N. Engl. J. Med.*, **308**, 129.

SLEE, P.H.Th.J., DE BRUIJN, E.A., DRIESSEN, O.M.J., HERMANS, J. & VAN OOSTEROM, A.T. (1983). Pharmacokinetics of the cytostatic drugs used in the CMF-regimen. *Anticancer Res.*, **3**, 269.

SLEE, P.H.Th.J., WILLEMZE, R., VAN OOSTEROM, A.T., LURVINK, E. & VAN DEN BERG, L. (1985). A comparison of two culture techniques: An *in vitro* and an *in vivo* tumour colony-forming assay. *Br. J. Cancer*, **52**, 713.

SLEE, P.H.Th.J., VAN OOSTEROM, A.T., VAN DEN BERG, L. & DE BRUIJN, E.A. (1986). The human tumour colony-forming assay using fresh specimens. *Neth. J. Med.*, **29**, 180.